# Limpet disturbance effects on barnacle recruitment are related to recruitment intensity but not recruit size

**DOI:** 10.7717/peerj.9190

**Published:** 2020-06-02

**Authors:** Julius A. Ellrich, Takefumi Yorisue, Kyosuke Momota

**Affiliations:** 1 Independent Researcher, Sankt-Josef-Straße, Koblenz, Germany; 2 Akkeshi Marine Station, Field Science Center for Northern Biosphere, Hokkaido University, Akkeshi, Hokkaido, Japan; 3 Integrative Aquatic Biology, Onagawa Field Center, Graduate School of Agricultural Science, Tohoku University, Onagawa, Miyagi, Japan; 4 Marine Environmental Information Group, Port and Airport Research Institute, Yokosuka, Kanagawa, Japan; 5 Institute of Natural and Environmental Sciences, University of Hyogo, Sanda, Hyogo, Japan; 6 Museum of Nature and Human Activities, Sanda, Hyogo, Japan

**Keywords:** Rocky intertidal ecology, Species interactions, Limpet bulldozing, Biological invasions, Introduced species, Biotic resistance

## Abstract

Intertidal limpets are important grazers along rocky coastlines worldwide that not only control algae but also influence invertebrates such as common barnacles. For instance, grazing limpets ingest settling barnacle cyprid larvae (hereafter cyprids) and push cyprids and barnacle recruits off the substrate. Such limpet disturbance effects (LDEs) can limit barnacle recruitment, a key demographic variable affecting barnacle population establishment and persistence. In this study, we examined limpet (*Lottia cassis*) disturbance to barnacle (*Chthamalus dalli*, *Balanus glandula*) recruitment on the Pacific coast of Hokkaido, Japan, as information on limpet-barnacle interactions from this region is missing. We investigated, for the first time, whether barnacle size and recruitment intensity influence LDEs on barnacle recruitment. Small barnacles may be less susceptible to LDEs than larger barnacles, because small size may reduce the propbability of limpet disturbance. Moreover, recruitment intensity can influence LDEs, as high recruitment can compensate for LDEs on barnacle recruitment density. In Hokkaido, *C. dalli* cyprids are smaller than *B. glandula* cyprids, and *C. dalli* recruitment is higher than *B. glandula* recruitment. Thus, we hypothesized that LDEs on *C. dalli* recruitment would be weaker than those on *B. glandula* recruitment. To test our hypothesis, we conducted a field experiment during which we manipulated limpet presence/absence on the interior surfaces of ring-shaped cages. After four weeks, we measured barnacle recruitment and recruit size on the interior surfaces of the cages and found negative LDEs on *C. dalli* and *B. glandula* recruitment and recruit size. As hypothesized, the LDEs on *C. dalli* recruitment were weaker than the LDEs on *B. glandula* recruitment. Additionally,* C. dalli* recruits were smaller than *B. glandula* recruits. However, the LDEs on *C. dalli* recruit size were as strong as the LDEs on *B. glandula* recruit size, indicating that the smaller* C. dalli* recruits are not less susceptible to LDEs than *B. glandula* recruits. Since *C. dalli* recruitment was higher than *B. glandula* recruitment, we propose that the higher *C. dalli* recruitment compensated for the LDEs on *C. dalli* recruitment. Our findings indicate that the detected differences in LDEs on barnacle recruitment are related to barnacle recruitment intensity but not recruit size.

## Introduction

Intertidal limpets (Patellogastropoda) are conspicuous grazers along coastlines worldwide (Branch, 2007; Heller, 2015). Therefore, limpet effects on benthic communities have received nearly global attention (Connell, 1961; Dayton, 1971; Branch, 1975; Menge, 1976; Denley & Underwood, 1979; Dungan, 1986; Iwasaki, 1993; Safriel, Erez & Keasar, 1993; Hodgson, 1999; Benedetti-Cecchi, 2000; Chan & Williams, 2003; Bazterrica et al., 2007; Zabin & Altieri, 2007). Research conducted along the North American Pacific coast has shown that limpets (*Lottia* spp.) not only control algae but also influence invertebrates, including the common barnacles *Chthamalus dalli* and *Balanus glandula* (Stimson, 1970; Dayton, 1971; Paine, 1981; Farrell, 1988; Miller & Carefoot, 1989; Farrell, 1991; Menge et al., 2010). Limpets can detach settled barnacle cyprid larvae (hereafter cyprids) and barnacle recruits from the substrate (Dayton, 1971; Miller & Carefoot, 1989; Menge et al., 2010). This process has been termed ‘limpet bulldozing’ since the limpets push the cyprids and recruits off the substrate (Dayton, 1971). Additionally, grazing limpets can ingest settled cyprids (Stimson, 1970; Dayton, 1971; Miller & Carefoot, 1989). Such limpet disturbance effects (LDEs) can limit barnacle recruitment (Dayton, 1971; Miller & Carefoot, 1989; Menge et al., 2010), which is the appearance of new barnacle individuals (i.e., recruits) that derive from settled and metamorphosed cyprids (Cole et al., 2011). Recruitment is a key demographic variable in barnacle population establishment (Alam et al., 2014) and persistence (Menge & Menge, 2013).

In this study, we examined LDEs on barnacle recruitment with a manipulative field experiment that used limpets (*Lottia cassis*) and barnacles (*C. dalli*, *B. glandula*) from the Pacific coast of Hokkaido, Japan, since information on limpet-barnacle interactions does not exist for this region. We investigated, for the first time, whether barnacle size and recruitment intensity influence LDEs on barnacle recruitment. Additionally, we evaluated whether disturbance effects by native *L. cassis* can contribute to biotic resistance, i.e., the ability of native species to limit invasions by introduced species (Yorisue, Ellrich & Momota, 2019), against the introduced *B. glandula* in Hokkaido.

*Lottia cassis* occurs along the Asian Pacific coast (Kussakin, 1977; Lin, Kong & Li, 2015; Okutani, 2017) and on the Pacific coast of California, USA (Okutani, 2017), and *C. dalli* is native to the northern Japanese and North American Pacific coasts (Drumm et al., 2016). *Balanus glandula* is native to the North American Pacific coast (Hiebert, Butler & Shanks, 2016), from where it was introduced, most likely through shipping, to the Pacific coast of Honshu, central Japan (Kado, 2003; Geller et al., 2008). On this coast, *B. glandula* has replaced several native barnacle species (Kado & Nanba, 2006). More recently, *B. glandula* spread to the Pacific coast of Hokkaido, northern Japan (Alam et al., 2014). In Hokkaido, the cyprid carapace length is ca. 250 µm in *C. dalli* and ca. 750 µm in *B. glandula* (Yorisue, personal observations). Thus, *C. dalli* cyprids are 67% smaller than *B. glandula* cyprids. Size information on early *C. dalli* and *B. glandula* recruits that resulted from recent cyprid metamorphoses is not available. However, observations in several barnacle species suggest that early recruits are slightly smaller than cyprids (Høeg et al., 2012; Maruzzo et al., 2012). In Hokkaido, *C. dalli* and *B. glandula* recruitment occurs from May to September, and *C. dalli* recruitment is typically higher than *B. glandula* recruitment since *B. glandula* established on this coast only recently (Alam et al., 2014; Yorisue, Ellrich & Momota, 2019).

Small barnacles may be less susceptible to LDEs than larger barnacles since small size may reduce the probability of disturbance (Paine, 1981). Thus, small barnacles may be less likely to be bulldozed (or grazed) by limpets than large barnacles. Moreover, recruitment intensity can influence LDEs (Menge et al., 2010), as high barnacle recruitment can compensate for LDEs on barnacle recruitment. Thus, we hypothesized that limpet disturbance would have weaker effects on *C. dalli* recruitment than on *B. glandula* recruitment. We tested our hypothesis on smooth substrate to exclude known rugosity influences on *C. dalli* and *B. glandula* settlement (Miller & Carefoot, 1989; Munroe, Noda & Ikeda, 2010) and to standardize LDEs on *C. dalli* and *B. glandula*.

## Materials & Methods

### Study system

We conducted this study on the Pacific coast of Hokkaido, Japan. Along this coast, limpets (*Lottia cassis*) and barnacles (*Chthamalus dalli*, *Balanus glandula*) are common organisms that co-occur in rocky mid-intertidal habitats (Nakaoka et al., 2006; Alam et al., 2014; Yorisue, Ellrich & Momota, 2019). *Lottia cassis* is patchily distributed (Yorisue, personal observations) and forages by grazing algae off the rocky substrate (Tsurpalo, 1995). Therefore, *L. cassis* can ingest small invertebrates (Tsurpalo, 1995).

### Manipulative field experiment

To test our hypothesis, we conducted a four-week field experiment in the harbour of Akkeshi Marine Station, Hokkaido University (latitude: 43.0212, longitude: 144.8368), in Akkeshi Bay during August and September 2017. At that time, *C. dalli* and *B. glandula* recruitment was relatively high compared to that in previous months. We established our experiment along the harbour wall, which sheltered the experiment from incoming waves. The harbour sea surface temperature (SST) and salinity (SSS) were measured daily. During the experiment, the SST and SSS were 17.3 ± 0.3 °C (mean ± SE) and 32.9 ± 0.1, respectively.

**Figure 1:**
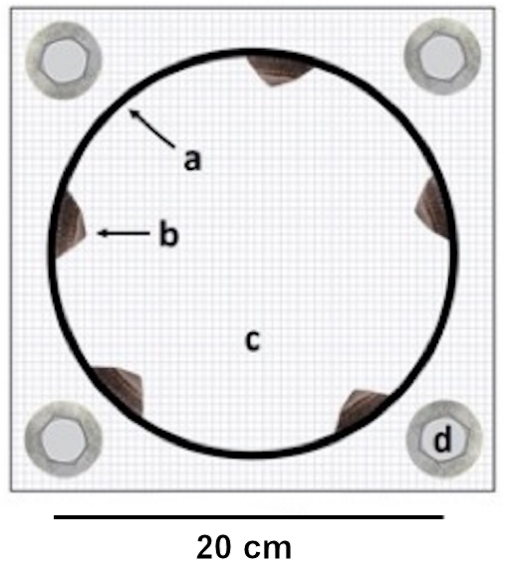
Experimental cage. Top view on an experimental cage showing (A) the PVC ring used to manipulate (B) limpet presence / absence on the interior ring surface, (C) the cage bottom mesh and (D) the washers and screws for cage attachment. The cage is displayed open to improve the view on the limpets but it remained sealed with a top mesh during the experiment. Barnacle recruit density was measured on the interior ring surface.

Each experimental unit consisted of a cage constructed of a smooth PVC ring (diameter: 20 cm, height: five cm, interior ring surface: 314 cm^2^) enclosed in plastic mesh (opening size: 0.5 cm × 0.5 cm). We chose this cage setup because previous field observations have indicated that *L. cassis*, *C. dalli* and *B. glandula* cyprids can attach to the ring. We manipulated limpet presence and absence by including five limpets (limpet cage) or no limpets (no-limpet cage) in the cages (Fig. 1). The limpet density in a limpet cage (1.6 limpets/100 cm^2^) was within the natural limpet density range(0–3 limpets/100 cm^2^) in Akkeshi Bay (Yorisue, personal observations). The average limpet shell length and width in the limpet cages were 2.5 ± 0.4 cm (±SE) and 1.9 ± 0.4 cm, respectively. Two separate one-factorial analyses of variance (ANOVAs) showed that limpet shell length and width did not differ among the limpet cages (shell length: F(6, 28) = 1.35, *p* = 0.270; shell width: F(6, 28) = 1.19, *p* = 0.341). The limpet shell length and width data met the assumptions for ANOVA (i.e., variance homogeneity and normality) as confirmed by Cochran’s C and Kolmogorov–Smirnov tests, respectively. We performed these analyses in Statistica 13.3 (Tibco Software Inc., Palo Alto, California, USA).

We performed our experiment in the mid-intertidal region according to the layout of a completely randomized block design (Gotelli & Ellison, 2004) by arranging 14 cages (i.e., seven limpet cages and seven no-limpet cages) along the harbour wall (see also Yorisue, Ellrich & Momota, 2019). We separated the cages with a 50 cm distance and attached them using plastic anchors, stainless steel screws and washers. We had previously removed all seaweeds (mainly *Chondrus yendoi*, *Saccharina japonica* and *Ulva* spp.) and adult barnacles (*C. dalli*, *B. glandula*), which constituted the most abundant organisms, dogwhelks (*Nucella lima*) and limpets (*L. cassis*) from the concrete harbour wall to prevent potential physical and/or chemical influences from these organisms on barnacle recruitment (Johnston & Strathmann, 1989; Miller & Carefoot, 1989; Beermann et al., 2013; Alam & Noda, 2016; Yorisue, Ellrich & Momota, 2019). On 8 August 2017, we began the experiment with pristine cages by placing the limpets into the limpet cages during low tide. The limpets readily attached to the interior ring surface when submerged by the following high tide but did not attach to the plastic mesh that enclosed the ring. On 6 September 2017, we collected all cages from the field and transported them to the lab. At that time, barnacle recruitment on the interior ring surface resembled barnacle recruitment on the natural substrates in Akkeshi Bay.

### Lab work

We measured barnacle recruitment by counting all *C. dalli* and *B. glandula* recruits on the interior ring surface (Fig. 1) and calculated *C. dalli* and *B. glandula* recruit density (i.e., barnacle recruit number/dm^2^). *Chthamalus dalli* and *B. glandula* recruits can easily be distinguished since brown *C. dalli* recruits are somewhat smaller than white *B. glandula* recruits (Hiebert, Butler & Shanks, 2016, see Results). As information on *C. dalli* and *B. glandula* recruit size from Hokkaido was not available, we additionally determined recruit basal shell diameter, a common measure of barnacle size, by measuring shell diameter along a straight line passing through the middle of the recruit rostrum and carina (Miller & Carefoot, 1989; Scrosati & Ellrich, 2019) using digital calipers. To do so, we randomly removed recruits from the interior ring surface of the cages using a stainless steel scraper and collected these recruits in 70% EtOH. We measured the basal shell diameter for 20 *C. dalli* recruits and 16 ± 1 (mean ± SE) *B. glandula* recruits from each cage since some cages had fewer than 20 *B. glandula* recruits. Using these size data, we calculated the average *C. dalli* and *B. glandula* basal shell diameter for each cage. As some limpets died during the experiment, we counted the number of limpet survivors at the end of the experiment. Finally, we examined whether any limpet recruits had occurred on the rings during the experiment.

### Data analyses

We examined the effects of limpet presence (two levels: limpet presence and absence) on barnacle recruitment and recruit size. We treated the blocks as random effects. For that, we conducted an analysis with generalized linear mixed models (GLMMs) with a Poisson distribution for barnacle recruit number and linear mixed models (LMMs) for barnacle recruit size using the ‘lme4’ package (Bates et al., 2015). We used GLMM analysis instead of ANOVA because our barnacle recruit density data had heterogeneous variances, as indicated by Cochran’s C tests. We performed these analyses in R 3.5.2 (R Core Team, 2018). Additionally, we calculated the sizes of the detected LDEs (Hedge’s g, Grizzard & Shaw, 2017) on *C. dalli* and *B. glandula* recruit density and *C. dalli* and *B. glandula* recruit size. Moreover, to investigate the potential interactions between *C. dalli* and *B. glandula*, we examined the relationships between *C. dalli* and *B. glandula* recruit density under limpet presence and absence. Finally, to evaluate whether limpet mortality influenced LDEs on barnacle recruit density and size, we examined the relationships between limpet survivors and *C. dalli* recruit density, *B. glandula* recruit density, *C. dalli* recruit size, and *B. glandula* recruit size. For these examinations, we used Pearson correlation analyses for barnacle recruit density and size and limpet survivor data after confirming normality with Kolmogorov–Smirnov tests (Dytham, 2011). We conducted these analyses in Statistica 13.3 (Tibco Software Inc., Palo Alto, California, USA).

## Results

### Barnacle recruitment and recruit size

**Figure 2:**
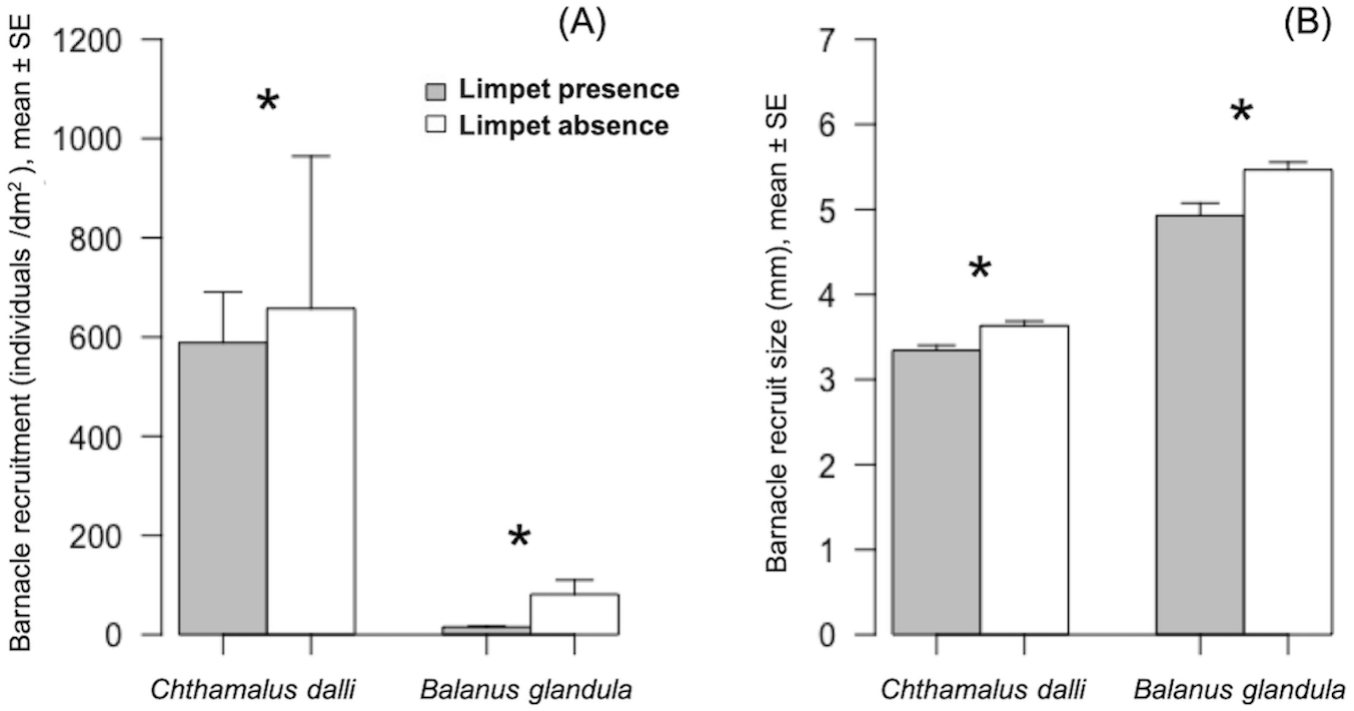
Barnacle (*Chthamalus dalli*, *Balanus glandula*) recruitment (A) and size (B) in limpet (*Lottia cassis*) presence and absence on the Pacific coast of Hokkaido, Japan in September 2017. Significant differences (*p* < 0.05) between two corresponding bars are indicated by an asterisk.

Limpet presence significantly limited *Chthamalus dalli* recruitment (GLMM: *χ*^2^ = 26.51, *p* < 0.001) by 10% and *Balanus glandula* recruitment (GLMM: *χ*^2^ = 251.61, *p* < 0.001) by 81% (Fig. 2A). The limpet disturbance effects (LDEs) on *C. dalli* recruitment (Hedge’s *g* = 0.114) were weaker than those on *B. glandula* recruitment (*g* = 1.218). In limpet absence, *Chthamalus dalli* recruitment was 12 times higher than *Balanus glandula* recruitment (Fig. 2A). Limpet presence had significant effects on *C. dalli* recruit size (LMM: *χ*^2^ = 14.08, *p* < 0.001) and *B. glandula* recruit size (LMM: *χ*^2^ = 14.61, *p* < 0.001). *Chthamalus dalli* and *B. glandula* recruits were 8% and 12%, respectively, smaller in limpet presence than in limpet absence (Fig. 2B). Correspondingly, the LDEs on *C. dalli* recruit size (*g* = 1.011) and *B. glandula* recruit size (*g* = 1.001) were similar. In limpet absence, *C. dalli* recruits were 32% smaller than *B. glandula* recruits (Fig. 2B).

### Barnacle-barnacle relationships, limpet recruitment and survival and limpet-barnacle relationships

There was no correlation between *C. dalli* and *B. glandula* recruit density in the presence of limpets (Pearson correlation: *r* = 0.48, *n* = 7, *p* = 0.276) and when limpets were absent (*r* = 0.17, *n* = 7, *p* = 0.723), indicating that there were no *C. dalli*-*B. glandula* interactions. No limpet recruits occurred in the cages during the experiment. On average, three limpets per cage (range: 1–4 limpets per cage) survived the experiment. There were no correlations between limpet survivors and barnacle recruit density (*C. dalli*: *r* =  − 0.06, *n* = 7, *p* = 0.893; *B. glandula*: *r* =  − 0.35, *n* = 7, *p* = 0.444) and no correlations between limpet survivors and barnacle recruit size (*C. dalli*: *r* =  − 0.02, *n* = 7, *p* = 0.969; *B. glandula*: *r* = 0.42, *n* = 7, *p* = 0.348), indicating that limpet mortality did not influence the LDEs on barnacle recruitment and size.

## Discussion

Using native limpets (*Lottia cassis*), native barnacles (*Chthamalus dalli*) and introduced barnacles (*Balanus glandula*) on the Pacific coast of Hokkaido (northern Japan), our manipulative field experiment showed that the LDEs on *C. dalli* recruitment are weaker than those on *B. glandula* recruitment. As some caged limpets died during our experiment, limpet density varied among the limpet cages. However, these experimental limpet densities still corresponded with the natural limpet density range in Akkeshi Bay (see Materials & Methods). Thus, despite the observed limpet mortality, our results are in line with previous findings from the North American Pacific coast, which showed that the LDEs on *C. dalli* recruitment are relatively weak compared to those on *B. glandula* recruitment (Dayton, 1971; Paine, 1981; Farrell, 1988; Miller & Carefoot, 1989; Farrell, 1991; Menge et al., 2010). Furthermore, our results resemble findings from Honshu (central Japan), which showed that the native limpets *Cellana toreuma, Siphonaria sirius* and *Scutellastra flexuosa* have only weak LDEs on recruitment in the native barnacle *Chthamalus challengeri* (Iwasaki, 1993), suggesting that LDEs on *Chthamalus* recruitment along the Japanese Pacific coast are relatively weak.

Corresponding with observations from the North American Pacific coast (Hiebert, Butler & Shanks, 2016), we detected that *C. dalli* recruits were smaller than *B. glandula* recruits. Additionally, concordant with our previous findings in Hokkaido (Yorisue, Ellrich & Momota, 2019), we found that *C. dalli* recruitment was more intense than *B. glandula* recruitment. Interestingly, we detected that *C. dalli* and *B. glandula* recruits were smaller in the presence of limpets than when limpets were absent, which suggests that limpets disturb early recruits. This notion is supported by the fact that limpet and snail disturbance effects on barnacle recruitment decrease with recruit size because growing barnacle recruits can reach a size refuge from LDEs (Dayton, 1971; Buschbaum, 2000; Denley & Underwood, 1979; Miller & Carefoot, 1989). Moreover, our results revealed that the LDEs on *C. dalli* and *B. glandula* recruit size were equally strong. These results suggest that although *C. dalli* cyprids and recruits are smaller than *B. glandula* cyprids and recruits, *C. dalli* is not less susceptible to LDEs than *B. glandula*. We propose that *C. dalli* recruitment (which was higher than *B. glandula* recruitment) compensated for the LDEs on *C. dalli* recruitment. We conclude that the weaker LDEs on *C. dalli* recruitment are related to *C. dalli* recruitment intensity but not to *C. dalli* size.

However, determining the exact mechanism underlying the detected differences in LDEs on *C. dalli* and *B. glandula* recruitment is beyond the scope of our study. In addition to recruitment intensity, this mechanism may involve cyprid metamorphosis speed (Menge et al., 2010) and recruit attachment strength (Miller, 1986). Working in Oregon (USA), Menge et al. (2010) showed that a greater proportion of *C. dalli* than *B. glandula* cyprids metamorphosed within a certain time. Thus, Menge et al. (2010) proposed that *C. dalli* resistance against LDEs is based on the ability of *C. dalli* cyprids to metamorphose quicker than *B. glandula* cyprids. Working in British Columbia (Canada), Miller (1986) found that *C. dalli* recruits more strongly attach to the substrate than similar-sized *B. glandula* recruits, suggesting that *C. dalli* recruits may be better protected from LDEs than *B. glandula* recruits. Data on cyprid metamorphosis speed and recruit attachment strength from Hokkaido do not exist but should be collected under varying barnacle recruitment intensity scenarios to examine the relative contributions of each of these three factors to the differential LDEs on *C. dalli* and *B. glandula* recruitment.

Understanding biotic resistance is a central goal of invasion biology (Stachowicz et al., 2002; Kimbro, Cheng & Grosholz, 2013). Recent research from Hokkaido revealed that native dogwhelks (*Nucella lima*) limit *B. glandula* abundance (Alam & Noda, 2016) and thus contribute to biotic resistance against *B. glandula*. These predatory snails prefer *B. glandula* as prey over *C. dalli* (Yorisue, Ellrich & Momota, 2019), likely as *B. glandula* is more nutritious (Palmer, 1983). Additionally, dogwhelk nonconsumptive effects, which are mediated through mucus-released chemical cues indicative of predation risk by dogwhelks (Johnston & Strathmann, 1989), limit *B. glandula* recruitment (Yorisue, Ellrich & Momota, 2019). Moreover, substrate pre-emption by *C. dalli* adults limits *B. glandula* abundance (Alam & Noda, 2016). However, our results show that *C. dalli* and *B. glandula* recruit density were not correlated, indicating that there are no interactions between *C. dalli* and *B. glandula* recruits under the examined recruit densities. As LDEs limited *B. glandula* recruitment by 81%, our results suggest that LDEs contribute to biotic resistance against *B. glandula*. This notion is supported by similar findings from the Argentinean Atlantic coast, which showed that LDEs from native limpets (*Siphonaria lessoni*, *Nacella magellanica*) can limit *B. glandula* recruitment (Bazterrica et al., 2007). Interestingly, Johnston & Strathmann (1989) showed that limpet (*Lottia scutum*) mucus-released chemical cues limit *B. glandula* recruitment in Washington (USA), likely as *B. glandula* cyprids seeking settlement move away when detecting such cues to reduce limpet disturbance risk. Thus, future research on biological resistance could examine whether (and by how much) limpet mucus cues limit *B. glandula* recruitment to evaluate whether such cues contribute to biotic resistance against *B. glandula*. Likewise, such research should quantify to what extent substrate rugosity, which can modify LDEs (Miller & Carefoot, 1989; Munroe, Noda & Ikeda, 2010), influences limpet biotic resistance against *B. glandula*.

##  Supplemental Information

10.7717/peerj.9190/supp-1 Supplemental Information 1 Sizes of barnacle recruit

10.7717/peerj.9190/supp-2 Supplemental Information 2 Number of barnacle recruits

10.7717/peerj.9190/supp-3 Supplemental Information 3 Supplemental Information 3

## Additional Information and Declarations

### Data Availability

The following information was supplied regarding data availability: The raw measurements are available in the Supplemental Files.
